# The long and winding road to translation for imaging biomarker development: the case for arterial spin labelling (ASL)

**DOI:** 10.1186/s41747-017-0004-7

**Published:** 2017-06-29

**Authors:** Xavier Golay

**Affiliations:** UCL Institute of Neurology, Queen Square 8-11, London, WC1N 3BG UK

**Keywords:** Quantitative evaluations, Biomarkers, Perfusion imaging, Calibration, Imaging, Phantoms

## Abstract

Radiology is facing many challenges nowadays, and certainly needs to keep up with the fast pace of developments taking place in this field. This editorial aims at drawing the attention of the reader to the current establishment of quantitative imaging biomarkers, in particular through the efforts of the Quantitative Imaging Biomarker Alliance (QIBA) from the Radiological Society of North America (RSNA), as well as the European Imaging Biomarker Alliance (EIBALL) from the European Society of Radiology (ESR). The case of arterial spin labelling (ASL) is used as an example of the long and winding road to translate a good imaging technique into a clinically relevant imaging biomarker.

## Key points

- Radiology is moving from pattern recognition by experts to a specialty based on the measurements of physical quantities
- In order to move from pattern recognition to the scientific assessment of measurement techniques, new paradigms need to be followed
- The two largest radiological associations in the world are leading the way through the establishment of dedicated committees working on this transformation
- Arterial spin labelling (ASL), an MRI technique used for the measurement of perfusion, is proposed as a demonstration of what is needed to transform an imaging technique into a quantitative imaging biomarker

## Introduction

Radiology is currently at a crossroads. Having been the first medical specialty to endorse the digital revolution, it is also the first to face the amazing opportunities, but also the profound threats, from artificial intelligence [1]. Such algorithms might one day be good enough to provide substantial help to radiologists everywhere around the world. In particular, they may be particularly good in helping them peruse the hundreds of sections typically provided by modern MRI or CT scanners for their reporting. Some fear, however, that they might be powerful enough to replace the radiologist altogether.

In parallel, another revolution is taking place, at a less mediatised rate but no less certainly than the AI one, and it has to do with quantification. Quantification in radiology starts with simple anatomical precision, and the well-known Response Evaluation Criteria In Solid Tumours criteria [2] used for assessment of treatment response in cancer are based on the premise that measurement of size can be made reproducibly over time, even if the patient is not scanned by the same machine functioning on the same software level. While these criteria are rather rough and simple, the issue of reproducibility and anatomical precision becomes already more challenging when MRI (or less frequently CT) is used to assess the slow reduction in grey matter taking place in dementia over time [3]. Precision becomes particularly crucial now that the new diagnostic criteria for dementia are based on an increase in the yearly rate of atrophy, at typically 2% for Alzheimer’s patients, with respect to the general population (0.5% per annum from the age of 40 years) [4, 5]. The issue only becomes more difficult to handle when such criteria are used as outcome measures in clinical trials, in which hundreds of patients need to be individually followed up, and for which precision needs to be maintained throughout the whole duration of the trial.

In this context, the pioneering natural history study called Alzheimer’s Disease Neuroimaging Initiative has established some of the necessary requirements needed in terms of quality assurance and reproducibility, as well as presence of artefacts [6]. Through its first results, published several years ago, extensive collaboration between basic scientists, statisticians and clinicians has allowed the development of the necessary standards for the use of imaging as an outcome measure in large trials. It thereby showed the necessity to use objects treated as reference standards across the various sites to ensure that no subtle drift was present, or that a scheduled software upgrade on a machine did not change the results dramatically [6].

Furthermore, the last 20–30 years have seen another revolution, beyond simple anatomical imaging, through which basic scientists and manufacturers have joined forces with radiologists to increase both the quality and the information content of the medical imaging equipment available. Quantitative imaging has gained a new meaning through the development of most physiological imaging techniques, be it, e.g. perfusion CT [7], Gd-based perfusion MRI [8], ASL [9] or diffusion-weighted imaging [10] and its many applications, from the assessment of white matter fibre tracts in the brain [11] to the detection and assessment of early changes in water diffusion in cancer [12]. Yet, following from 30 years of development leading these techniques to be widely used in most oncological examinations everywhere in the body, they are today still mostly interpreted in a semiqualitative way by radiologists around the world. This is happening in the face of a large body of evidence indicating that the quantitative measures themselves obtained by many of these techniques could serve as early indicators of the presence of disease or indeed as biomarkers of response to treatment [13–16]. In addition, these techniques offer the added advantage of being usable as translational biomarkers between late preclinical studies involving animal models and first-in-man studies, thereby providing early indications of its potential therapeutic power. As such, it is hoped that the use of quantitative physiological imaging as translational biomarkers by basic scientists and clinicians alike might one day allow a shortening of the time to market of new therapeutics. More importantly for this community, it will naturally increase the participation by radiology departments in clinical trials, and ensure its more frequent position as a leading partner.

So, as radiology, like many other medical specialties, moves towards a more evidence-based approach, and as quantification becomes an ever more important part of its practice, it becomes necessary for it to become more precise, and with precision comes the need to become more scientific. In particular, the implementation of quantitative anatomical and physiological imaging requires the use of very strict rules based on metrology, the science dealing with measurement. This is particularly difficult for radiology, owing to the differences between the acquisition and analysis tools available on the market, as well as the independent activities of the clinicians. It is, therefore, absolutely necessary for the field to move forward to increase the collaboration between basic scientists and clinicians in order to overcome the hurdles linked with the development of quantitative imaging biomarkers. Understanding the seriousness of these issues, the Radiological Society of North America decided in 2007 to establish the Quantitative Imaging Biomarker Alliance (QIBA) as a means to unite researchers, health care professionals, and industry stakeholders to advance the use of quantitative imaging in general [17].

Through QIBA, scientists, clinicians and mathematicians hope to validate quantitative imaging biomarkers, based on metrological practices such as identification and characterisation of the sources of error. In addition, a detailed analysis of the entire imaging chain will need to be undertaken, from acquisition to processing, to be able to establish the presence or not of a bias along the entire measurement procedure. Here again, estimation of a bias size is generally made through the use of objects serving as ‘gold standards’ or benchmarks for the measurements done. These objects are generally called phantoms and their role will, therefore, be more and more important within the growing field of quantitative radiology.

In Europe as well, responding to the urgent need to promote the development of imaging biomarkers, the European Society of Radiology (ESR) has created a standing subcommittee from its Research Committee, called the European Imaging Biomarker Alliance (EIBALL). This committee aims at promoting the development of biomarkers within the realm of the ESR, and has recently joined forces with the European Institute of Biomedical Imaging Research to start working on Europe based projects in this matter. In particular, the EIBALL Committee has recently joined forces with QIBA to work on the first-ever EIBALL-QIBA project on the development of a new quantitative imaging biomarker, based on the ASL perfusion measurement technique.

## A typical case of biomarker development: ASL

The case of ASL is rather typical for a quantitative imaging technique. Started in the early 1990s [9], the method underwent many technical improvements through a decade of developments by MRI physicists and engineers. This left the field in the early 2000s with many independent implementations, leading the major manufacturers to pick and choose one each differently as a work-in-progress package [18]. In addition, at that time, no clear consensus existed in terms of quantification, and every researcher and the few clinicians interested in the applications of this method were left with a rather daunting choice for quantification models, each providing a slightly different answer, depending on its underlying hypotheses [19].

Therefore, the community had to do something to try to sort out the two main issues plaguing the field: (1) the plethora of acquisition techniques and (2) the lack of consensus on the quantification method. In addition, nobody even knew whether this technique was reproducible, apart from a handful of volunteers being scanned repeatedly at single institutions. Indeed, it took nearly 20 years between the first publication on rats to the first large test-retest study of one of the numerous techniques [20].

Thus, in 2009, a core group of researchers and clinicians gathered together at a first meeting in London, and decided to establish the ASL Network (http://www.asl-network.org). This group has since met on regular occasions primarily at meetings of the International Society for Magnetic Resonance in Medicine (ISMRM) and, from 2011 to 2015, it was supported by a European Commission-funded COST Action to try and establish the use of ASL in dementia [18].

One of the main achievements of this action has been the publication, together with the Perfusion Study Group of the ISMRM, of a position paper on the current state of ASL, the so-called ‘ASL White Paper’, indicating clearly what sequence was thought to be providing the best signal-to-noise ratio and what quantification method needed to be employed [21]. Remarkably, this paper, in addition to its 14 coauthors, was also officially endorsed by over 230 people, representing a large proportion of basic scientists and clinicians active at that time. In addition to this landmark paper, numerous other studies tackled the problem of reproducibility and difference in perfusion maps obtained by the different manufacturers (e.g. [22]). This led all major manufacturers to slowly change their implementation to the preferred version from the ASL White Paper.

It is, therefore, the right time now to engage with the process of establishing a QIBA profile, and it is great news that members of the EIBALL Committee have agreed to bring it forward. Within its tasks, the committee will need to implement further longitudinal studies and to refine claims to establish exactly how quantitative assessment of cerebral perfusion can shape the future of neurological and neuroradiological research and applications. The future now seems bright, with the potential use of quantitative perfusion in stroke, dementia, and brain tumours, as well as in neuroinflammation and other neurological conditions!

Within this context, it seems that many of the upcoming activities linked in particular to quantitative imaging biomarker development and validation, such as the case in ASL, as well as its implementation within clinical practice, fall exactly within the target publication of this very journal, *European Radiology Experimental*.

## Abbreviations

ASL: Arterial spin labelling
EIBALL: European Imaging Biomarker Alliance
ESR: European Society of Radiology
ISMRM: International Society for Magnetic Resonance in Medicine
QIBA: Quantitative Imaging Biomarker Alliance

## References

[1] Jha S, Topol EJ (2016). Adapting to artificial intelligence: radiologists and pathologists as information specialists. JAMA.

[2] Therasse P, Arbuck SG, Eisenhauer EA (2000). New guidelines to evaluate the response to treatment in solid tumors. European Organization for Research and Treatment of Cancer, National Cancer Institute of the United States, National Cancer Institute of Canada. J Natl Cancer Inst.

[3] O’Brien JT, Paling S, Barber R (2001). Progressive brain atrophy on serial MRI in dementia with Lewy bodies, AD, and vascular dementia. Neurology.

[4] Dubois B, Feldman HH, Jacova C (2010). Revising the definition of Alzheimer’s disease: a new lexicon. Lancet Neurol.

[5] Scahill RI, Frost C, Jenkins R (2003). A longitudinal study of brain volume changes in normal aging using serial registered magnetic resonance imaging. Arch Neurol.

[6] Jack CR, Bernstein MA, Fox NC (2008). The Alzheimer’s Disease Neuroimaging Initiative (ADNI): MRI methods. J Magn Reson Imaging.

[7] Nabavi DG, Cenic A, Craen RA (1999). CT assessment of cerebral perfusion: experimental validation and initial clinical experience. Radiology.

[8] Rosen BR, Belliveau JW, Vevea JM, Brady TJ (1990). Perfusion imaging with NMR contrast agents. Magn Reson Med.

[9] Williams DS, Detre JA, Leigh JS, Koretsky AP (1992). Magnetic resonance imaging of perfusion using spin inversion of arterial water. Proc Natl Acad Sci U S A.

[10] Wesbey GE, Moseley ME, Ehman RL (1984). Translational molecular self-diffusion in magnetic resonance imaging. II. Measurement of the self-diffusion coefficient. Investig Radiol.

[11] Mori S, Crain BJ, Chacko VP, van Zijl PC (1999). Three-dimensional tracking of axonal projections in the brain by magnetic resonance imaging. Ann Neurol.

[12] Takahara T, Imai Y, Yamashita T, Yasuda S, Nasu S, Van Cauteren M (2004). Diffusion weighted whole body imaging with background body signal suppression (DWIBS): technical improvement using free breathing, STIR and high resolution 3D display. Radiat Med.

[13] Galban CJ, Chenevert TL, Meyer CR (2009). The parametric response map is an imaging biomarker for early cancer treatment outcome. Nat Med.

[14] Padhani AR, Liu G, Koh DM (2009). Diffusion-weighted magnetic resonance imaging as a cancer biomarker: consensus and recommendations. Neoplasia.

[15] Willett CG, Duda DG, di Tomaso E (2009). Efficacy, safety, and biomarkers of neoadjuvant bevacizumab, radiation therapy, and fluorouracil in rectal cancer: a multidisciplinary phase II study. J Clin Oncol.

[16] Wolk DA, Detre JA (2012). Arterial spin labeling MRI: an emerging biomarker for Alzheimer’s disease and other neurodegenerative conditions. Curr Opin Neurol.

[17] Sullivan DC, Obuchowski NA, Kessler LG (2015). Metrology standards for quantitative imaging biomarkers. Radiology.

[18] Golay X, Guenther M (2012). Arterial spin labelling: final steps to make it a clinical reality. MAGMA.

[19] Petersen ET, Zimine I, Ho YC, Golay X (2006). Non-invasive measurement of perfusion: a critical review of arterial spin labelling techniques. Br J Radiol.

[20] Petersen ET, Mouridsen K, Golay X, all named co-authors of the QUASAR test-retest study (2010). The QUASAR reproducibility study, Part II: results from a multicentre Arterial Spin Labeling test-retest study. NeuroImage.

[21] Alsop DC, Detre JA, Golay X (2015). Recommended implementation of arterial spin-labeled perfusion MRI for clinical applications: a consensus of the ISMRM Perfusion Study Group and the European Consortium for ASL in Dementia. Magn Reson Med.

[22] Mutsaerts HJ, van Osch MJ, Zelaya FO (2015). Multi-vendor reliability of arterial spin labeling perfusion MRI using a near-identical sequence: implications for multi-center studies. NeuroImage.

